# Immunogenicity is preferentially induced in sparse dendritic cell cultures

**DOI:** 10.1038/srep43989

**Published:** 2017-03-09

**Authors:** Aikaterini Nasi, Vishnu Priya Bollampalli, Meng Sun, Yang Chen, Sylvie Amu, Susanne Nylén, Liv Eidsmo, Antonio Gigliotti Rothfuchs, Bence Réthi

**Affiliations:** 1 Department of Microbiology, Tumor and Cell Biology, Karolinska Institutet, Stockholm, Sweden; 2 Department of Medicine, Karolinska University Hospital and Karolinska Institutet, Solna, Sweden; 3 Department of Medicine, Science for Life Laboratory, Karolinska Institutet, Solna, Sweden

## Abstract

We have previously shown that human monocyte-derived dendritic cells (DCs) acquired different characteristics in dense or sparse cell cultures. Sparsity promoted the development of IL-12 producing migratory DCs, whereas dense cultures increased IL-10 production. Here we analysed whether the density-dependent endogenous breaks could modulate DC-based vaccines. Using murine bone marrow-derived DC models we show that sparse cultures were essential to achieve several key functions required for immunogenic DC vaccines, including mobility to draining lymph nodes, recruitment and massive proliferation of antigen-specific CD4+ T cells, in addition to their TH1 polarization. Transcription analyses confirmed higher commitment in sparse cultures towards T cell activation, whereas DCs obtained from dense cultures up-regulated immunosuppressive pathway components and genes suggesting higher differentiation plasticity towards osteoclasts. Interestingly, we detected a striking up-regulation of fatty acid and cholesterol biosynthesis pathways in sparse cultures, suggesting an important link between DC immunogenicity and lipid homeostasis regulation.

Dendritic cells (DCs) represent a heterogeneous group of antigen presenting cells that play an essential role in the initiation of the adaptive immune responses through inducing naive T cell activation in secondary lymphoid tissues. However, several alternative fates of T cell differentiation can be promoted by DCs due to their own developmental divergence into functionally specialized subsets and due to an ability to fine-tune their functional repertoire in response to a variety of signals such as microbial compounds, cytokines or metabolites.

Various strategies have been developed to modulate antigen-specific immune responses with the help of *ex vivo* generated autologous DCs^1^,^2^. DC-based anticancer vaccines could potentially deliver tumour-associated antigens to lymphoid tissues and induce the activation of antigen-specific CD4+ and CD8+ T cells that can home to neoplastic lesions and mediate tumour regression. DC vaccines have been applied to more than 3000 patients suffering melanoma, prostate cancer, glioma or renal cell cancer and the results of these studies indicated increased median survival in most vaccinated cohorts^1^. However, only a small proportion of treated individuals displayed detectable tumour regression and discordance has been frequently noted between immunological and clinical responses with detectable tumor-specific immune responses often contributing to little impact on the overall disease burden^1^.

The small number of individuals who respond favourably to DC vaccinations indicates the need for developing more immunogenic DC vaccines and to dissect the reasons underlying the highly variable clinical responses. Previous findings have highlighted several mechanisms that contributed to DC vaccine efficiency including higher IL-12 production^3^,^4^, efficient co-stimulatory signals^5^, stronger induction of antigen-specific TH1 responses^6^,^7^,^8^ or lower regulatory T cell numbers in the tumor tissue^6^,^9^. Other parameters, such as the site of injection, the number of injected DCs or the number of DCs reaching the T cell zone of lymph nodes are also critical for DC vaccine efficiency^10^,^11^,^12^. It has been shown that only a small fraction of the injected DCs reach the draining lymph node^10^,^11^,^12^ and increasing DC mobility improved survival in gliobastoma patients^13^.

Interestingly, a significant heterogeneity in DC cell surface markers and functional characteristics has been detected not only *in vivo* but also among *ex vivo* generated DCs. Coexisting CD1a^+^CD14^−^ and CD1a^−^CD14^low^ populations developed from blood monocytes in presence of GM-CSF and IL-4 or from CD34^+^ hematopoietic progenitors cultured with GM-CSF and Flt3-L, and these two DC subsets presented unique functional characteristics^14^,^15^. In particular, the CD1a^+^CD14^−^ population was superior in inducing TH1 polarization and cytotoxic T lymphocyte (CTL) killer activity as compared to the CD1a^−^CD14^low^ counterpart. Importantly, the CD1a^+^/CD1a^−^ DC ratio varied greatly among blood donors, suggesting that a developmental heterogeneity might influence immunogenicity in individual DC vaccine preparations^14^.

We have recently described a cell concentration-dependent differentiation switch in DC cultures, which further contributed to the diversity of DC phenotypes *in vitro*. By increasing cell concentration in the early phase of development we reduced CCR7-dependent chemotaxis and the ability to induce TH1 responses in human monocyte-derived DCs (MoDCs) and we observed elevated IL-10 production^16^,^17^. Such effects could potentially compromise the ability of DC vaccines to induce immunity against cancer or pathogens, however; further *in vivo* experiments are necessary to understand whether the density-dependent endogenous breaks could influence DC-based therapies.

In the present work we demonstrate that DC differentiation in sparse cultures promoted several functional characteristics required for immunogenic DC vaccines, namely the ability to migrate to secondary lymphoid organs and increase lymph node cellularity, in addition to the induction of massive proliferation and TH1 polarization of antigen-specific CD4+ T cells. We analysed the transcriptional programs underlying DC differentiation in dense or sparse cultures and unravelled several characteristic immunoregulatory pathways in the unique density-dependent lineages. Interestingly, we detected an increased expression of several genes involved in fatty acid and cholesterol biosynthesis in the more immunogenic DC preparations, obtained from sparse cultures, suggesting a potential role of lipid homeostasis regulation in promoting the development of DCs with an immunogenic phenotype.

## Results

### Endogenous DC regulatory pathways observed in sparse and dense cell cultures

**Figure 1:**
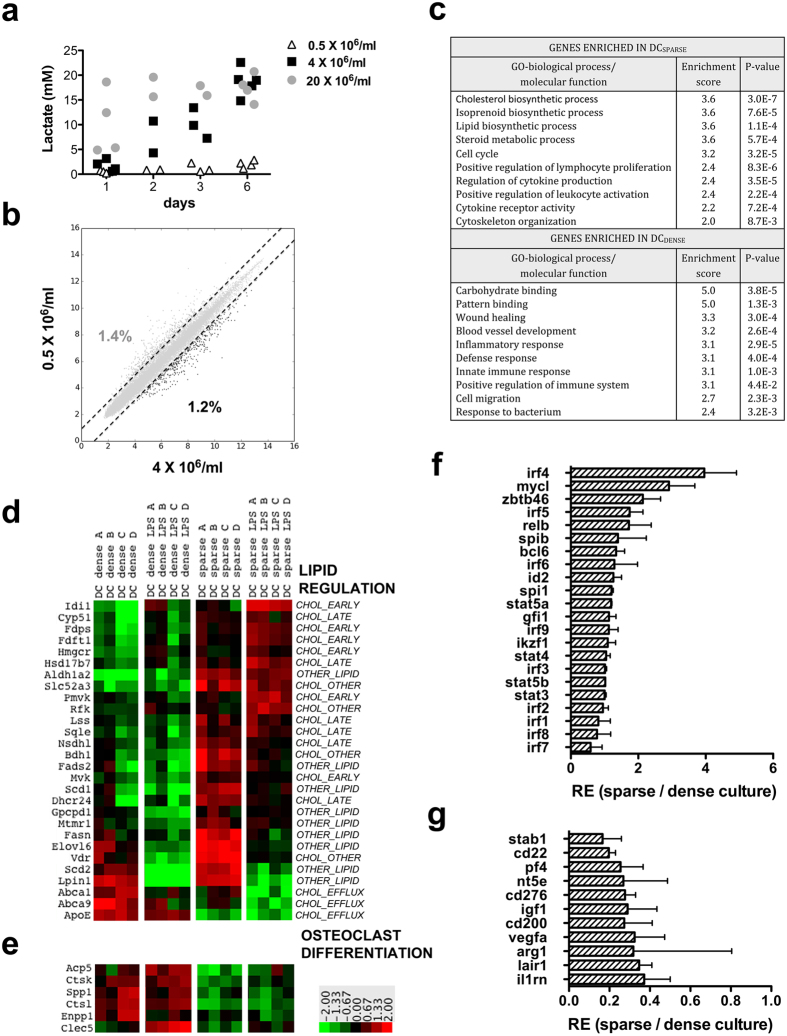
Cell culture density alters BMDC development. (**a**) BMDC cultures were established using 0.5, 4 and 20 × 10^6^/ml initial cell concentrations and lactate levels were monitored in the supernatants. (**b**) Gene expressions are compared between BMDCs developing at 0.5 × 10^6^ and 4 × 10^6^ cells/ml densities. Dashed lines indicate 2-fold difference in transcription levels between the two DC types, the numbers represent the frequency of genes with >2-fold higher expression level in sparse or dense cultures. (**c**) Gene ontology enrichment analysis of highly expressed genes in sparse and dense DC cultures. The heatmaps show the expression pattern of set of genes involved in cholesterol and fatty acid homeostasis (**d**) and osteoclast differentiation (**e**) in the different BMDC preparations before and after a 4-hour LPS treatment. Genes involved in lipid biosynthesis were categorised according to their roles in early mevalonate pathway (CHOL_EARLY), late stages of cholesterol production and cholesterol modifications (CHOL_LATE), indirect modulation of cholesterol biosynthesis (CHOL_OTHER), cholesterol efflux processes (CHOL_EFFLUX) or other lipid biosynthetic processes regulated through cholesterol-sensitive gene expressions (OTHER_LIPID). Colours indicate relative log2-transformed expression levels compared to median values for each visualized genes. Relative expression levels are also shown for a group of transcription factors that regulate DC differentiation (**f**) and for immunosuppressive compounds (**g**). Average expression levels and SD values are calculated from 4 parallel experiments.

We have previously shown that lactic acid accumulates in dense human MoDC cultures, and induces a cell concentration-dependent reprogramming of cytokine production and cell surface markers^16^. To monitor whether immunizations with antigen-loaded DCs could be affected by cell culture density, we generated mouse BMDCs using cell concentrations that were associated with different lactate levels in the supernatants (0.5 × 10^6^, 4 × 10^6^ and 20 × 10^6^ bone marrow cells/ml initial cell concentrations, Fig. 1a). Notably, in these BMDC cultures we observed comparable lactate concentrations as previously, in human MoDC cultures established at a range of 0.125 × 10^6^–2 × 10^6^ monocytes/ml^16^, indicating similar glycolytic environments in the mouse and human cultures in spite of different precursor cell compositions and densities in the two models.

To identify unique differentiation clues associated with sparse or dense cultures we compared gene expressions in BMDCs generated using 0.5 × 10^6^/ml and 4 × 10^6^ cells/ml initial densities. Approximately 2.6% of the detected transcripts were differentially expressed in the two DC lineages with 1.4 and 1.2% of the genes showing >2-fold higher expression in sparse and dense cultures, respectively (Fig. 1b). Gene ontology enrichment analysis of the data set revealed a remarkable association of genes involved in lipid biosynthesis with sparse DC cultures (Fig. 1c,d). Several of these genes encode key enzymes of the early mevalonate-isoprenoid pathway, which contribute to both cholesterol biosynthesis and to post-translational modifications providing cellular localisation signals to signalling components (Fig. 1d). Other enzymes participate in later stages of cholesterol biosynthesis and cholesterol modifications or, alternatively, can influence cholesterol homeostasis through indirect mechanisms, including flavoenzyme regulation or transport processes. Lastly, a group of fatty acid and retinoic acid biosynthesis genes were highly expressed in sparse cultures, which are all strictly regulated by a cholesterol-sensitive manner through transcription factors of the sterol regulatory element-binding protein (SREBP) family. LPS-mediated DC activation induced a rapid reprogramming of cholesterol-related gene expressions with pronounced upregulation of several cholesterol biosynthesis genes, however, only in DCs originated from sparse cultures. On the other hand, genes encoding the cholesterol efflux proteins ABCA1 and ABCA9 and the cholesterol transport molecule ApoE were expressed at higher level in dense cultures, suggesting limited cholesterol retention in these cells (Fig. 1d). It is noteworthy, that another set of genes involved in synthesis, transport or modification of diverse lipid products followed different expression patterns, several of them being up-regulated in both DC types upon LPS treatment (Supplementary Figure 1).

Another set of genes highly expressed in sparse DC cultures suggested an increased capacity of the cells to communicate with other immune cells and to induce T cell activation (Fig. 1c). These included genes encoding MHC class II molecules and their transcription regulator CIITA, co-receptor molecules CD24a, CD40, CD80, CD54, ICOSL, TNFSF4 and TNFSF8, the activation marker CD83, cytokines and cytokine receptors including TNF, IL2RA, IL1R1, IL1R2, IL12RB, IL18RAP, LIFR and the NF-kB components NF-kB1 and c-Rel (Supplementary Figure 1). Genes highly expressed in dense cultures encoded several pattern recognition molecules including lectins, Toll-like receptors and the LPS binding protein (LBP), as well as compounds involved in extracellular matrix interactions (fibronectin, matrix metalloproteinase 19), blood coagulation (factor III and V), complement and complement binding proteins (C1Rl, CFH, C5AR1) and several chemokines (Fig. 1c, Supplementary Figure 1). Interestingly, a small group of genes encoding osteoclast related proteins (Acid phosphatase 5, Cathepsin K and L, Osteopontin and C-type lectin domain family 5) were also associated with dense DC cultures (Fig. 1e), highlighting an important similarity with the human MoDC system where dense cultures increased differentiation plasticity towards osteoclasts^16^.

Differentiation switch points for myeloid lineages are often regulated by key transcription factors, which facilitate the segregation of macrophages and DCs or the functional specialization of diverse DC subsets^18^. DCs obtained from dense or sparse cultures were characterized by comparable expression of several DC regulatory transcription factors suggesting at least partially similar transcription machinery at different cell culture densities (Fig. 1f). On the other hand, *MycL* and *Irf4*, which modulate T cell priming and the induction of different helper T cell lineages by DCs^19^,^20^,^21^, were expressed at higher levels in sparse cultures indicating potentially unique features of antigen presentation in the two DC types. This hypothesis was further supported by the accumulation of a diverse set of mediators involved in immunosuppressive mechanisms among the genes highly expressed in dense DC cultures^22^,^23^,^24^,^25^,^26^,^27^,^28^,^29^,^30^, including *Lair1*, *Arg1*, *Vegfa*, *Igf1*, *Stab1*, *Pf4*, *Nt5e*, *Cd276*, *Cd200* (Fig. 1g). In summary, we identified a few characteristic sets of genes, which showed notable association with sparsity (genes linked to cholesterol homeostasis, inflammation and DC-T cell interactions) or with high cell concentrations (pattern recognition, osteoclast differentiation, immunosuppression) in DC cultures.

### Immunogenic phenotype of LPS-activated DCs required sparse DC cultures

**Figure 2:**
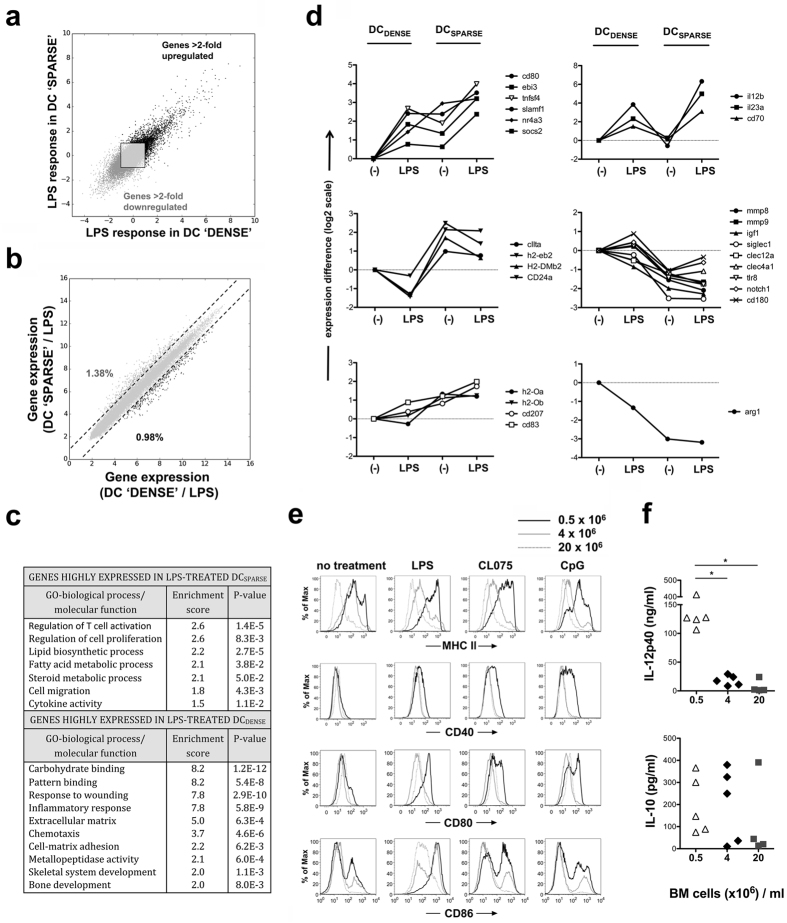
Cell culture density-dependent DC phenotypes following LPS stimulation. (**a**) LPS-mediated gene expression changes are compared for BMDCs obtained from dense and sparse cultures (4 × 10^6^ and 0.5 × 10^6^ bone marrow cells/ml, respectively). Genes up- or down-regulated at least 2-fold are visualized in black or dark grey colours, respectively. (**b**) Gene expressions following 4-hour LPS treatment in DCs obtained from dense or sparse cultures. Dashed lines represent 2-fold expression difference, the numbers indicate the frequency of genes with > 2-fold higher expression level in the different DC types. (**c**) Gene ontology enrichment analysis of highly expressed genes in the two DC types following LPS activation. (**d**) Relative expression of a group of immune response-associated genes clustered based on similar expression patterns. Average expression levels were calculated from four independent samples for each condition. Gene expression differences after LPS stimulation reflected baseline differences, except for the group of *IL12b*, *IL23a*, *CD70* genes where different expression levels were observed only after LPS treatment. (**e**) Cell surface expression of MHC II, CD40, CD80 and CD86 upon in *in vitro* activation with LPS, CL075 and CpG for 24 h was measured by flow cytometry. Representative results of three independent experiments are shown. (**f**) IL-12p40 and IL-10 concentrations in BMDC supernatants were measured by ELISA following 24-hour LPS stimulation. The symbols represent cytokine levels measured in different experiments.

LPS activation induced relatively comparable transcriptional programs in DCs generated using 0.5 × 10^6^ or 4 × 10^6^ bone marrow cells/ml densities (Fig. 2a) and only a small amount of genes, 1.38 and 0.98% of the detected transcripts, were expressed at higher level in the cells originated in sparse or dense cultures, respectively (Fig. 2b). Gene ontologies associated with dense or sparse DC cultures following LPS treatment were in alignment with the analysis of non-activated DCs, as reflected by several lipid biosynthesis and T cell activation-related genes highly expressed when DCs were obtained from sparse cultures and genes associated with pattern recognition, tissue homeostasis (extracellular matrix, wound healing) and innate immune functions were highly expressed in DCs derived from dense cultures (Fig. 2c and Supplementary Figure 1). In fact, gene expression differences between LPS-activated DC types mostly originated from the baseline expression pattern that characterized non-activated DCs, with expression levels slightly modulated by LPS-induced up- or downregulations in sets of similarly expressed genes (Fig. 2d). In this respect, genes for IL-12p40 (*Il12b*), IL-23p19 (*Il*-*23a*) and CD70 (*Cd70*) showed a unique expression profile, as higher expression levels in DCs originated from sparse cultures were the sole result of a more pronounced LPS-induced upregulation in these cells. In addition to a different transcriptional profile, we observed higher cell surface expression of the MHC II, CD40, CD80 and CD86 molecules in DCs obtained from sparse cultures, in the presence of various TLR ligands (Fig. 2e). Moreover, high levels of IL-12 were induced by LPS selectively in DCs developing in sparse cultures (Fig. 2f). IL-10 production, on the contrary, was independent of cell culture densities. Notably, MHC II and co-stimulatory molecules were expressed similarly in human monocyte-derived DCs developing at different densities, but IL-10 production was strongly associated with dense MoDC cultures^16^. The differences between the human and mouse experimental DC systems suggested complex and at least partially different density-dependent pathways operating in the two models. This hypothesis was further suggested by the findings that a glucose-galactose replacement in the cell culture medium, which reduced lactic acid levels and boosted IL-12 production in dense human MoDC cultures, had no effect on the IL-12 production in mouse BMDCs (n = 4, data not shown).

### DCs developing in sparse cultures display superior mobility towards draining lymph nodes

**Figure 3:**
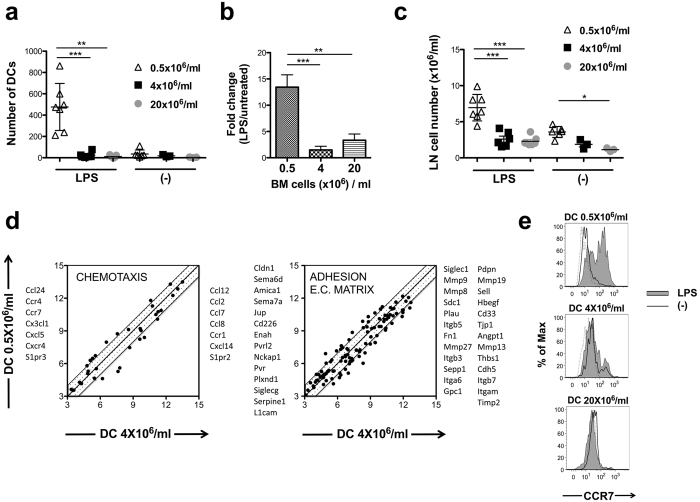
Cell culture sparsity improves BMDC mobility *in vivo*. BMDCs were generated using various cell culture densities (0.5, 4 and 20 × 10^6^/ml). The cells were CFSE-labelled and incubated in the presence or absence of LPS for 3 hours. The DCs were then washed and injected to the footpad of B6 mice using 10^6^ cells/injection. On day 3 the CFSE+ DC numbers were analysed in the draining popliteal lymph nodes using flow cytometry (**a**). Numbers of LPS-activated DCs are also presented following normalization with background migration (non-activated DCs) to exclude potential differences in individual DC preparations (**b**). In addition to migrating DCs, the total lymph node cell numbers were also analyzed in the draining popliteal lymph nodes (**c**). Expression levels of various genes associated with pathways related to chemotaxis and cell adhesion/extracellular matrix interactions are shown for LPS-activated DCs that were obtained from dense or sparse cultures (**d**). Genes characterized by an at least 2-fold expression in either of the DC types are listed by the respective sides of the dot plots. CCR7 expression was measured using flow cytometry on BMDCs generated using different cell culture densities after 24 h incubation with or without LPS (**e**). Dotted lines represent staining with isotype control antibodies. Representative results of two independent experiments are shown. *p < 0.05, **p < 0.01, ***p < 0.001.

The ability of DCs to relocate from the periphery to the draining lymph nodes is essential for the initiation of an immune response and it can strongly influence the immunogenicity of DC vaccines^11^,^13^. Therefore, we next investigated how cell concentrations during DC development influence the *in vivo* mobility of DCs injected to the footpad of C57Bl6 mice. A significantly increased number of LPS-activated DCs migrated to the lymph nodes when the cells were obtained from sparse cultures as compared to dense DC cultures or to non-activated DCs (Fig. 3a,b). At the same time, popliteal LNs from mice receiving DCs from sparse cultures exhibited a significantly increased cellularity, even in the absence of LPS-mediated activation signals, as compared to dense DC cultures (Fig. 3c). Such lymph node enlargements in response to sparse culture-derived DCs were the consequence of predominantly lymphocytic cell accumulation, with 22.4 + 3.0% of the LN cells being CD4^+^ and 24.2 + 2.5% being CD8^+^ T cells (n = 4). To delineate the molecular bases of the different mobility and lymphocyte recruiting potential we compared the expression of genes involved in chemotaxis and cell adhesion-related pathways in LPS-activated DCs obtained from dense or sparse cultures. Several of these genes were expressed at different levels in the two DC types (Fig. 3d), which might lead to different migratory and lymphocyte-attractant capacity in the cells. In addition, different levels of inflammation and immune activation induced by the two DC types could also modulate cellular migration. Notably, several genes involved in DC mobility were expressed at higher level in DCs obtained from sparse cultures, including S1pr3 that has been shown to promote mouse DC migration towards Sphingosine 1-phosphate^31^, Semaphorin 7A, which increases DC mobility through regulating extracellular matrix interactions^32^ and CCR7 and CXCR4, two chemokine receptors that can target DCs into peripheral lymph nodes. Using flow cytometry we detected CCR7 expression on >50% of DC obtained from sparse cultures, following the activation of the cells with LPS, whereas little or no CCR7 expression was observed on DCs obtained from dense cultures (Fig. 3e). Although detectable CCR7 expression on the cell surface might not be a prerequisite for DC mobility to draining lymph nodes^33^, our results suggested an important link between CCR7 expression levels and the different mobility of DCs obtained from sparse or dense cultures.

### **I**ncreased BMDC culture density impacts negatively on T cell activation, proliferation and Th1 polarization *in vitro*

**Figure 4:**
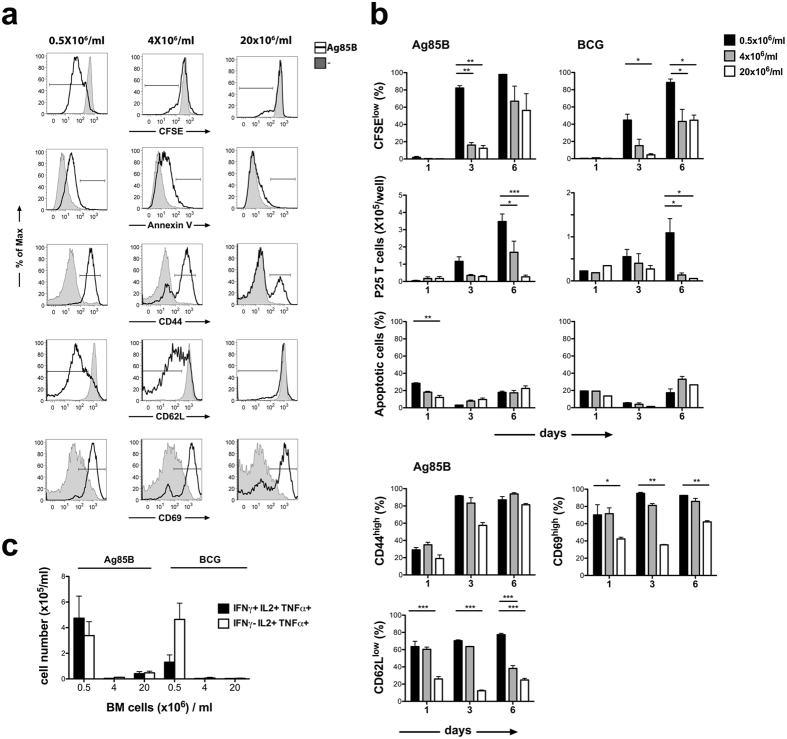
Sparsity in BMDC cultures promotes antigen-specific T cell activation and proliferation *in vitro*. BMDCs were loaded with Ag85B and activated by LPS or were treated with BCG and then co-cultured with CFSE-labeled P25 T cells. (**a**) Representative histograms indicate CFSE dilution in proliferating P25 T cells, apoptosis measured by Annexin V binding, and the modulation of CD44, CD62L and CD69 expression on P25 T cell by day 3 in the presence of DCs obtained from dense or sparse cultures. (**b**) Frequencies of CFSE low P25 T cells, their absolute number, the proportion of Annexin V-binding apoptotic cells and the expression of CD44, CD62L and CD69 on P25 T cells are shown at different time points of the DC - T cell co-cultures. (**c**) Concentration of IFNγ+IL2+TNFα+ or IFNγ-IL2+TNFa+ T cells were measured by flow cytometry on day 6 of DC - T cell co-cultures. **P < 0.01, ***P < 0.001.

We examined the influence of cell concentration on the ability of the developing DCs to induce antigen-specific T cell activation, proliferation and cytokine production *in vitro*. BMDCs developing in dense or sparse cultures were loaded with the mycobacterial Ag85B peptide or incubated with living BCG and were thereafter used as antigen presenting cells for P25 TCR-tg T cells. We detected a significantly higher level of T cell proliferation by day 3 of the culture when DCs were obtained from sparse cultures, as compared to higher densities (Fig. 4a,b). The difference in CFSE dilution decreased in the following 6 days, especially when DCs were loaded with peptide antigen, due to the proliferating cells outnumbering non-dividing ones in all conditions. Nevertheless, a significantly higher number of antigen-specific T cells accumulated in the presence of DCs obtained from sparse cultures indicating more efficient T cell activation in these cultures. T cell apoptosis was similar in all culture conditions, indicating that the different P25 T cell numbers reflected different rates of proliferation and not the variable maintenance of the cells (Fig. 4a,b). Upregulation of CD69 and CD44 and the downregulation of CD62L occurred similarly at DC culture densities 0.5 × 10^6^ and 4 × 10^6^ BM cells/ml although the CD62low phenotype was more stabile when DCs were obtained from sparse culture (Fig. 4b). Altogether, our data indicated that DCs developing at all tested cell culture densities were able to trigger naive T cell activation, however, a higher level of T cell proliferation in presence of DCs derived from sparse cultures contributed to the accumulation of a larger population of antigen-specific T cells.

We analysed the cytokine profile of P25 T cells incubated for 6 days in presence of antigen-loaded DCs generated in dense or sparse cultures and observed high numbers of IFNγ^+^IL-2^+^TNF^+^ triple positive and IL-2^+^TNF^+^ double positive P25 T cells when T cell activation occurred in presence of DCs that developed in sparse cultures and, in line with the proliferation data, very few cytokine producing cells were observed when T cell were incubated with DCs obtained from dense cultures (Fig. 4c).

### Sparsity in developing DC cultures increases T cell priming efficiency *in vivo*

**Figure 5:**
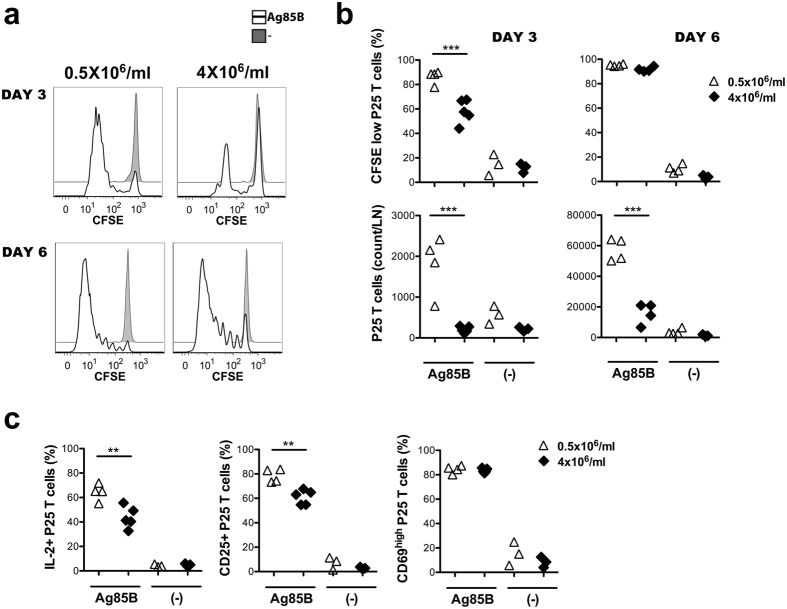
Antigen specific T cell activation *in vivo* in response to BMDCs obtained from sparse or dense cell cultures. (**a**) The histograms show CFSE dilution in proliferating CD45.2 + P25 T cells isolated from draining lymph nodes of CD45.1+ mice that had been previously immunized by BMDCs generated by using the cell culture densities of 0.5 or 4 × 10^6^ BM cells/ml. (**b**) Frequencies of CFSE low CD45.2 + P25 T cells and the P25 T cell numbers in the draining lymph nodes are shown in the different animals immunized by LPS-activated DCs loaded or not with the Ag85B peptide. Representative results of two independent experiments are shown. (**c**) The frequencies of IL-2-producing, CD25+ and CD69 + P25 T cells were analysed using LN cells of mice immunized with the different BMDC preparations. **P < 0.01, ***P < 0.001.

The ability of DCs obtained from dense or sparse cultures to prime P25 TCR-Tg T cells was next investigated *in vivo*. BMDCs were generated from CD45.1^+^ mice, loaded with Ag85B_240–254_, stimulated with LPS and subsequently injected into the footpad of naïve CD45.1^+^ recipients. CFSE-labelled, naïve P25 TCR-Tg cells were injected i.v. into the same animals 24 hours earlier. On day 3 we detected a significantly higher P25 T cell proliferation in the popliteal lymph nodes in the animals that received DC vaccines generated in sparse cultures (Fig. 5a,b). In line with this finding, IL-2 production and the expression of CD25 that confers high affinity IL-2 binding to activated T cells were more pronounced in P25 T cells activated by DCs obtained from sparse cultures, whereas the activation marker CD69 was induced similarly by the two DC types (Fig. 5c). On day 6 we observed similar CFSE staining profile in T cells activated by the different DCs, however, a somewhat higher proportion of non-proliferating T cells in case of dense DC cultures indicated less efficient T cell priming in these mice (Fig. 5a,b). In fact, P25 T cell numbers in draining lymph nodes were significantly higher both at day 3 and day 6 in mice that receives DC vaccines generated in sparse cultures, potentially reflecting the cumulative effects of the more efficient recruitment and priming of the antigen-specific T cells in these mice.

## Discussion

DCs can direct the immune response to either tolerogenic or immunogenic directions due to their substantial functional variability, indicating the importance of understanding how DC-based vaccines can be programmed to induce the optimal types of responses. The appearance of different phenotypes in the same DC cultures suggests alternative differentiation fates even in the presence of the same differentiation signals^14^,^15^. Heterogeneity of the precursor populations or endogenous mechanisms acting between the developing cells might both contribute to the variability of DC lineages, however, little is known about the exact mechanisms underlying DC diversity in cell cultures. We have previously described an unexpected and vigorous modulation of human MoDC functions already at very early stages of their development by an endogenous, cell culture density-dependent mechanism^16^. Dense cultures, although frequently used for MoDC differentiation, were associated with little migratory potential, high IL-10 and low TNF, IL-23 and IL-12 production *in vitro*, strongly suggesting a reduced immunogenicity for such DC vaccine preparations. We have shown that lactic acid, a side product of glycolytic energy production, accumulated in dense MoDC cultures and played an important role in reducing the development of IL-12-producing CD1a^+^CD14^−^ DCs^16^, similarly as it has previously been shown in MoDCs developing in tumour microenvironment^34^. Although our present results suggested a role for endogenous lactic acid in DC modulation only in the case of human cells, we have observed strikingly different DC differentiation when comparing dense and sparse cultures in the mouse BMDC model as well. Sparsity was essential to achieve DCs that efficiently migrate to the draining lymph nodes from the injection sites and induce a rapid accumulation of lymphocytes, even in the absence of antigen-specific T cell activation. The superior chemotactic effect was most probably achieved by the combined effect of the higher DC numbers arriving to the lymph node and the more efficient production of various inflammatory mediators in addition to T cell recruiting chemokines by DCs obtained from sparse cultures. In addition to increased mobility, DCs obtained from sparse cultures were more efficient in priming naive CD4^+^ T cell activation and inducing massive proliferation. The results clearly indicated the importance of sparse cultures when DCs are generated with the aim of inducing immunity against cancer or pathogens. On the other hand, the immunomodulatory effects of DCs obtained from dense cultures are most probably compromised by their limited mobility and little efficiency in inducing T cell activation.

An important factor contributing to DC reprogramming in dense cultures might be hypoxia, which potentially increases in line with cell concentration. Previous studies applying persistently low oxygen concentration have revealed extensive modifications in DC development, both in human and murine experimental systems, which are partially overlapping with features observed in dense DC cultures, however, important differences can also be noted^35^. Murine BMDCs developing at low oxygen tension decreased their IL-12 production similarly to the effect of dense cell cultures, however, low oxygen tension also resulted in increased expression of costimulatory and MHC molecules and in higher CCR7 levels and DC mobility, which are in striking contrast to dense BMDC cultures^36^. It is noteworthy that have observed a modest upregulation of a group of hypoxia-regulated genes (hexokinase, VEGF, IGF-1 encoding genes, Fig. 1g and Supplementary Figure 1) in dense cultures, which might influence metabolism and various functions in developing DCs. However, the key differences in protein expressions and cell migration between murine DCs developing under hypoxic conditions or in dense cell cultures suggests that hypoxia alone might not be responsible for the altered DC functions at different cell concentrations.

A variety of molecules associated with antigen presentation and T cell activation were highly expressed in sparse DC cultures, whereas DC obtained from dense cultures were characterized by gene expressions associated with immunosuppressive mechanisms, potentially contributing to their limited ability of inducing T cell activation, and diverse pathways related to innate immune functions (pattern recognition, complement system and blood coagulation). The ability of DCs to develop into osteoclasts has been demonstrated both *in vitro* and *in vivo*^37^,^38^. In the human MoDC system, dense cultures increased the trans-differentiation of DCs to osteoclasts^16^ and, in line with these results, we detected highly expressed genes encoding several osteoclast-associated molecules in dense BMDC cultures. Notably, gene expression differences observed between dense and sparse DC cultures were largely preserved following LPS-mediated activation, indicating persisting functional consequences induced by the density-dependent differentiation signals.

Interestingly, a group of genes involved in fatty acid and cholesterol biosynthesis were highly expressed in sparse cultures, whereas the genes encoding cholesterol efflux proteins were expressed at higher level in DCs obtained from dense cultures. It is intriguing to speculate that an increased lipid biosynthesis could contribute to higher mobility and T cell activating potential in *ex vivo* generated DCs. Previous studies have indeed suggested a possible link between lipid synthesis and DC immunogenicity. Protein farnesylation and geranylgeranylation require intermediates from cholesterol biosynthesis and these modifications regulate membrane association and function of several key signalling components including Ras, RhoA, Rac1 or Cdc42^39^. In addition, new membrane production is required to fuel an increased protein synthesis, transport and secretion in activated DCs by allowing the expansion of ER and golgi membranes^40^ and the production of cholesterol-rich microdomains, which accumulate the MHC and co-stimulatory molecules and play important role in synapse formation between DCs and T cells^41^. It has been previously shown that inhibition of cholesterol biosynthesis in monocyte-derived DCs using statins resulted in a reduced expression of MHC II and co-stimulatory molecules and a weakened ability of the DCs to stimulate T cell proliferation^42^. Fatty acid and cholesterol availability is regulated sensitively by the coordinated action of the sterol regulatory element binding proteins and the LXR transcription factors^43^. It is noteworthy that DCs obtained from sparse cultures were characterized by higher expression of various genes regulated by the SREBP transcription factors, which suggests a scenario where an increased membrane production in the DCs would induce the upregulation of the biosynthetic pathway-components through depleting free cholesterol in the cells and consequently increasing SREBP activity. Further studies are needed to understand how fatty acid and cholesterol biosynthesis influence DC vaccines, however, the remarkable association of these pathways with DC mobility and antigen presenting capacity *in vivo* suggests a key role for lipid neogenesis in producing immunogenic DC vaccine preparations.

In summary we described in the present work a cell culture density-dependent differentiation switch in cultured DCs, which might play a crucial role in determining the immunogenicity of therapeutic DC vaccine preparations. Sparsity in DC cultures promoted the acquisition of a transcriptional program that suggested commitment to antigen presentation and robust T cell activation by the DCs. Our results also highlighted that fatty acid and cholesterol biosynthesis-related pathways might provide important opportunities for functional modulation or monitoring of therapeutic DC preparations.

## Methods

### Mice and Ethical permits

CD45.1+ mice on a C57BL/6 background (B6.SJL/Ptprc^a^) originally from Charles River Laboratories, and C57BL/6 mice were both maintained at the MTC breeding unit, Karolinska Institutet, Stockholm, Sweden. P25 TCR-Tg mice^44^ crossed to ECFP RAG-1^−/−^ background^33^ were kindly provided by Dr. Ronald Germain, NIAID, NIH. All animals were maintained under specific pathogen-free conditions at MTC. Both male and female mice between 8 and 12 weeks old were used. Animals were housed, handled and all experiments were performed in accordance to the directives and guidelines of the Swedish Board of Agriculture, the Swedish Animal Protection Agency, and Karolinska Institutet (djurskyddslagen 1988:534; djurskyddsförordningen 1988:539; djurskyddsmyndingeheten DFS 2004:4). The experiments were approved by the Stockholm North Animal Ethics Council.

### Bone marrow-derived DC cultures

Bone marrow-derived DC (BMDC) cultures were established using RPMI 1640 medium (endotoxin level <0.04 U/ml) complemented with 10% FCS (Life technologies, Carlsbad, CA, USA), antibiotics and recombinant mouse GM-CSF (Life technologies) as previously described^45^. CD11c^+^ BMDCs were enriched to 97–99% using magnetic separation (Miltenyi Biotech, Auburn, CA, USA) on day 6 and used for further experiments. BMDC were activated using LPS (250 ng/ml for *in vitro* experiments and 100 ng/ml for adoptively transferred DCs), CL075 (1 μg/ml) or CpG (2.5 uM), all from Invivogen (San Diego, CA, USA). In some experiments glucose-free RPMI 1640 medium (Life technologies) was complemented with 2 g/L D-galactose (Sigma Aldrich, St. Louis, MO, USA).

### *In vitro* T cell activation

CD11c^+^ BMDCs were loaded with 0.5 μM Ag85B_240–254_ for 3 h and then activated by LPS for 2 hours. Alternatively, the cells were incubated with Bacillus Calmette-Guerin (BCG) strain Pasteur at a multiplicity of infection (MOI) of 1 for 5 h. Naïve P25 TCR-Tg cells were labelled with 0.25 μM CFSE (Invitrogen, Carlsbad CA, USA) and co-cultured with DCs using 1:4 DC:T cell ratio. Proliferation and apoptosis were monitored using flow cytometry on days 1, 3 and 6. Cytokine production was measured after reactivation of the cell *in vitro* using 50 ng/ml PMA and 1 μg/ml ionomycin (Sigma Aldrich, St. Louis, MO, USA) in presence of GolgiPlug (BD Biosciences, San Jose, CA, USA) for 5 hours. Cell numbers were calculated using Countbright beads (Invitrogen, ThermoFisher Scientific, Waltham, MA, USA).

### Analysis of DC migration and antigen presentation *in vivo*

BMDCs were labelled with CFSE (3 μM) followed by 3-hour incubation in the presence or absence of LPS. The cells were then washed and adoptively transferred into the hind footpad of mice (1 × 10^6^ DCs/mouse). Three days later the draining popliteal LNs were isolated, single cell suspensions were prepared as previously described^33^ and the CFSE^+^ DCs numbers were measured using Countbright beads for flow cytometry. To assess CD4^+^ antigen-specific T-cell responses, LN cells from naïve P25 TCR-Tg RAG-1^−/−^ mice were labelled with CFSE (1 μM) and injected intravenously in the tail vein of naïve, congenic CD45.1^+^ recipients (0.1 or 1 × 10^6^ cells/mice). Higher cell numbers were used for mice receiving DCs without cognate antigens to ensure detection of the transferred T cells. BMDCs were pre-incubated with or without 0.5 μM Ag85B_240–254_ for 3 hours, and then treated by LPS for 2 hours. For intracellular cytokine measurements, cells from draining LNs were stimulated *ex vivo* for 6 hours with 10 μM Ag85B_240–254_ peptide in the presence of GolgiPlug.

### Flow cytometry

FITC-labelled anti-IA/IE, PE-labelled anti-Vβ11, anti-TNFα, anti-CD45.2, anti-CD40, APC-conjugated anti-CD80, anti-IFNγ, anti-CD69, PeCy7-labelled anti-CD11c, PercypCy5.5-labeled anti-CD44, anti-IL-2 and APC-Cy7-conjugated anti-CD62L were obtained from BD Pharmingen (San Diego, CA, USA). PE- labelled anti-CD86, PE-efluor 610-labelled anti-CD25 and Pacific blue conjugated-Annexin V were from Biolegend (San Diego, CA, USA). Alexa 405-conjugated anti-CD4 was obtained from CALTAG lab (Buckingham, UK). APC-conjugated anti-IA/IE and PE-labelled anti-CCR7 were obtained from eBioscience (San Diego, CA, USA). All stainings were performed in presence of mouse Fc Block (BD Biosciences). Dead cells were stained using the Live/Death detection kit with a near-infrared dye (Invitrogen). The samples were analysed using CyAn ADP Analyser (Beckman Coulter, Brea, CA, USA) and the data were analysed using FlowJo version 9.2 (Tree Star inc, Ashland, Oregon, USA).

### Microarray analysis

For RNA isolation we used RNase easy kit (Qiagen, Hilden, Germany) complemented with DNase I treatment. Gene expression analysis using the Affymetrix gene chip array was performed according to the instruction of the manufacturer at the Bioinformatics and Expression Analysis facility, Karolinska Institutet. Microarray data are available at the Array Express repository (www.ebi.ac.uk/arrayexpress) under accession number E-MTAB-4614. After normalisation and log2 transformation we included genes for pathway analysis that showed at least 2-fold mean expression difference between any of the sample groups. For heatmaps, individual log2-transformed gene expression levels were centred using median values and genes were then clustered hierarchically based on uncentred Pearson correlation and average linkage. The Java Treeview software version 1.1.6r4^46^ was used for visualization. Gene ontology enrichment and pathway overrepresentation analyses were performed using the David gene ontology website and the ConsensusPathDB mouse database using information from Reactome, Mouse Cyc and KEGG^47^,^48^.

### Cytokines and lactic acid measurements in BMDC supernatants

IL-12/23p40 was measured by sandwich ELISA as previously described^49^, IL-10 was measured using ELISA from eBiosciences. Lactic acid levels were analysed using the glycolysis cell-based assay kit from Cayman Chemical (Ann Arbor, MI, USA).

### Statistical analysis

The data were analyzed using one-way Anova and Tukey’s post-test with the help of Graph Pad Prism version 5.0a, unless stated otherwise in the text.

### Ethical approval

Mice used in the study were housed and handled, and all experiments were performed, in accordance with the directives and guidelines of the Swedish Board of Agriculture, the Swedish Animal Protection Agency, and Karolinska Institutet (djurskyddslagen 1988:534; djurskyddsförordningen 1988:539; djurskyddsmyndingeheten DFS 2004:4). The experiments were approved by the Stockholm North Animal Ethics Council.

## Additional Information

**How to cite this article:** Nasi, A. *et al*. Immunogenicity is preferentially induced in sparse dendritic cell cultures. *Sci. Rep.* **7**, 43989; doi: 10.1038/srep43989 (2017).

**Publisher's note:** Springer Nature remains neutral with regard to jurisdictional claims in published maps and institutional affiliations.

## Supplementary Material

Supplementary Figure 1
