# Supplementary material for: Immunogenicity is preferentially induced in sparse dendritic cell cultures
Source: Sci Rep. 2017 Mar 9;7:43989. doi: 10.1038/srep43989 (PMC5343661; doi:10.1038/srep43989)
Supplement: Supplementary Figure 1 [file srep43989-s1.doc]

**SUPPLEMENTARY INFORMATION**

**Immunogenicity is preferentially induced in sparse dendritic cell cultures**

Aikaterini Nasi^a^, Vishnu Priya Bollampalli^a^, Meng Sun^b^, Yang Chen^c^, Sylvie Amu^a^, Susanne Nylén^a^, Liv Eidsmo^b^, Antonio Gigliotti Rothfuchs^a^ and Bence Réthi^b^

*^a^Department of Microbiology, Tumor and Cell Biology, Karolinska Institutet, Stockholm, Sweden*

*^b^Department of Medicine, Karolinska University Hospital and Karolinska Institutet, Solna, Sweden*

*^c^Department of Medicine, Science for Life Laboratory, Karolinska Institutet, Solna, Sweden*

SIGNAL TRANSDUCTION

**
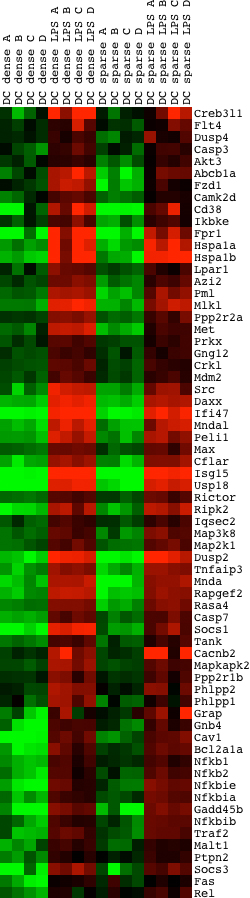

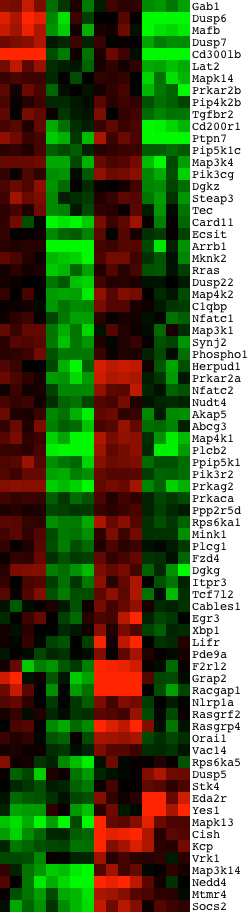

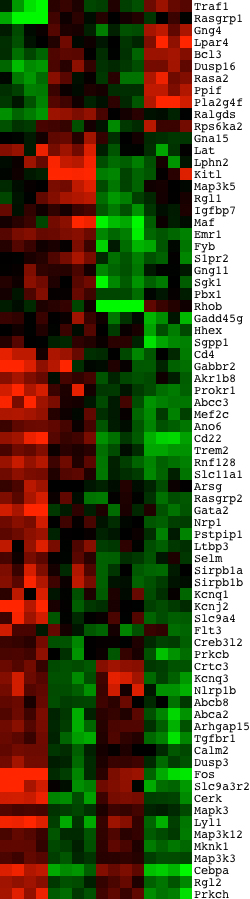
**

**
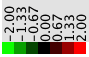
**

ADHESION CYTOKINE & CHEMOKINE ANTIGEN


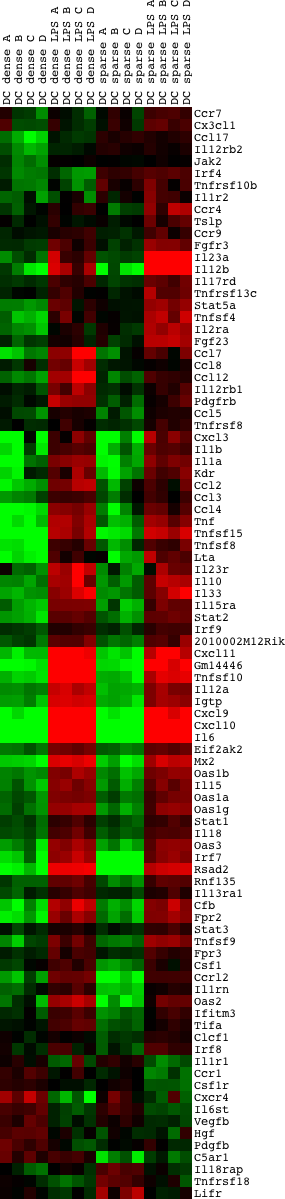

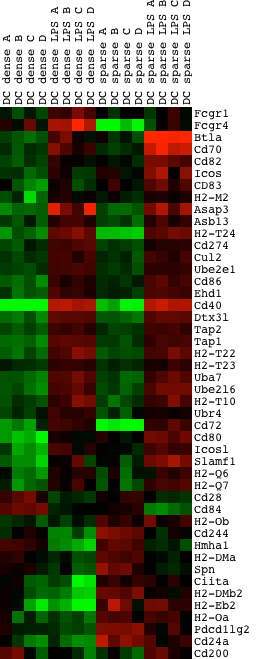
 & E.C. MATRIX PATHWAYS PRESENTATION


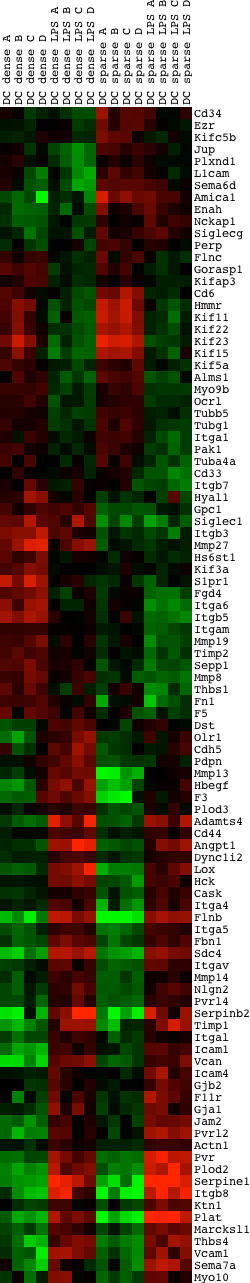


PATTERN

RECOGNITION


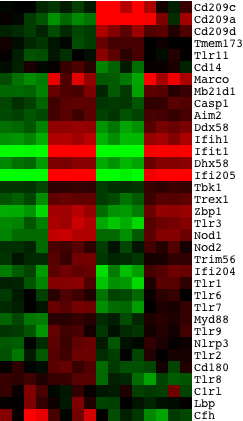


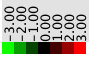


LIPID CARBOHYDRATE

METABOLISM METABOLISM

**
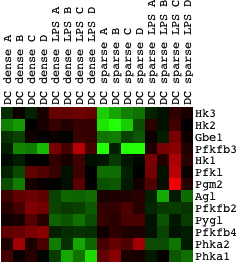

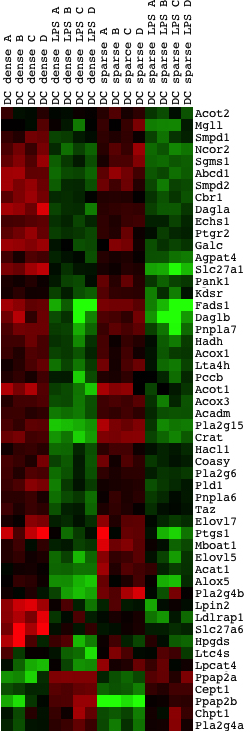
**

**
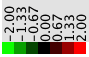
**

**Supplementary figure 1**. Gene expressions are compared between BMDCs developing at 0.5x10^6^ and 4x10^6^ cells/ml densities from 4 independent experiments. Genes characterized by at least 2-fold difference between mean expression levels of any of the samples groups were used for pathway analysis using ConsensuspathDB. The log2-transformed expression levels are related to the median values for each gene and the data is clustered based on individual expression patterns. Lipid metabolism heatmap is complementary to Figure 1d.
